# Supplementary material for: Comparative Assessment of Quantification Methods for Tumor Tissue Phosphoproteomics
Source: Anal Chem. 2022 Jul 26;94(31):10893–906. doi: 10.1021/acs.analchem.2c01036 (PMC9366746; doi:10.1021/acs.analchem.2c01036)
Supplement: Supplementary file 1 — ac2c01036_si_001.pdf [file ac2c01036_si_001.pdf]

Comparative assessment of quantification methods for tumor tissue phosphoproteomics

Yang Zhang[1-3], Benjamin Dreyer[4], Natalia Govorukhina[1], Alexander M. Heberle[2,3], Saša Končarević[5], Christoph Krisp[4], Christiane A. Opitz[6,7], Pauline Pfänder[6,8], Rainer Bischoff[1], Hartmut Schlüter[4], Marcel Kwiatkowski[2,9,10,*], Kathrin Thedieck[2,3,11,*], Peter L. Horvatovich[1,*]

[1]Department of Analytical Biochemistry, Groningen Research Institute of Pharmacy, University of Groningen, 9713 AV Groningen, The Netherlands

[2]Institute of Biochemistry and Center for Molecular Biosciences Innsbruck, University of Innsbruck, 6020 Innsbruck, Austria

[3]Laboratory of Pediatrics, Section Systems Medicine of Metabolism and Signaling, University of Groningen, University Medical Center Groningen, 9713 AV, Groningen, The Netherlands

[4]Section/Core Facility Mass Spectrometry and Proteomics, Institute of Clinical Chemistry and Laboratory Medicine, University Medical Center Hamburg-Eppendorf, Martinistraße 52, 20246 Hamburg, Germany

[5]Proteome Sciences R&D GmbH & Co. KG, Altenhöferallee 3, 60438 Frankfurt/Main, Germany

[6]Metabolic Crosstalk in Cancer, German Consortium of Translational Cancer Research (DKTK) & German Cancer Research Center (DKFZ), Heidelberg, Germany

[7]Department of Neurology, National Center for Tumor Diseases, University Hospital Heidelberg, Heidelberg, Germany

[8]Faculty of Bioscience, Heidelberg University, Heidelberg, Germany

[9]Department of Molecular Pharmacology, Groningen Research Institute for Pharmacy, University of Groningen, Groningen 9700 AD, The Netherlands

[10]Groningen Research Institute for Asthma and COPD, University Medical Center Groningen, University of Groningen, Groningen 9700 AD, The Netherlands.

[11]Department of Neuroscience, School of Medicine and Health Sciences, Carl von Ossietzky University Oldenburg, 26129 Oldenburg, Germany

*Corresponding authors

Tables of content

Supplementary figures -- S2

Supplementary tables -- S8

Supplemental experimental section --S10

References --S13

Supplementary figures

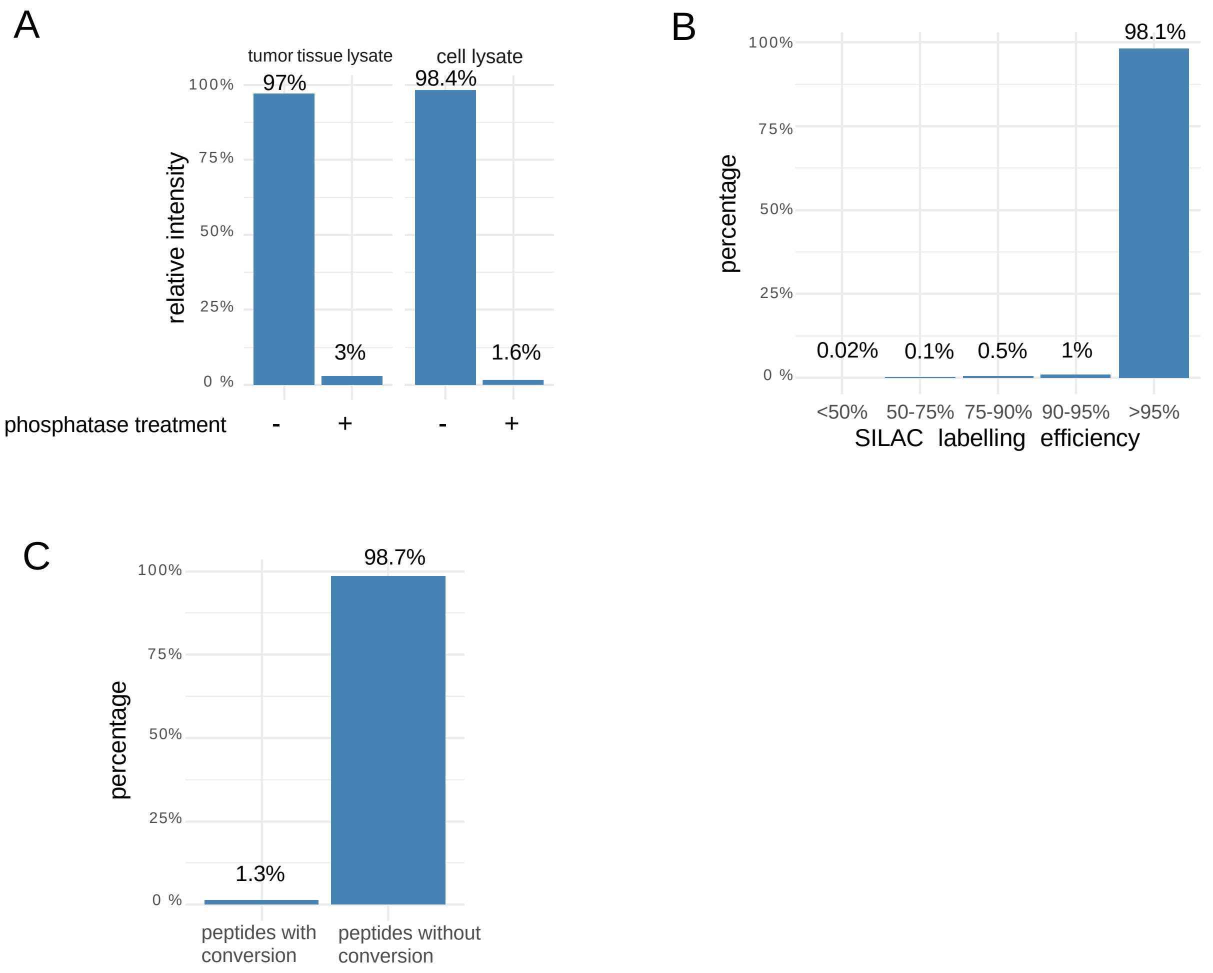

Figure S1: Dephosphorylation efficiency, SILAC labelling efficiency and arginine-to-proline conversion. (A) Relative intensities of all identified phosphopeptides without and with alkaline phosphatase treatment. The total relative intensities of tumor tissue lysates and cell lysates were 100%, respectively. (B) Bar plot showing the distribution of the identified peptides according to their SILAC labelling efficiency. (C) Relative intensities of peptides with and without arginine-to-proline conversion.

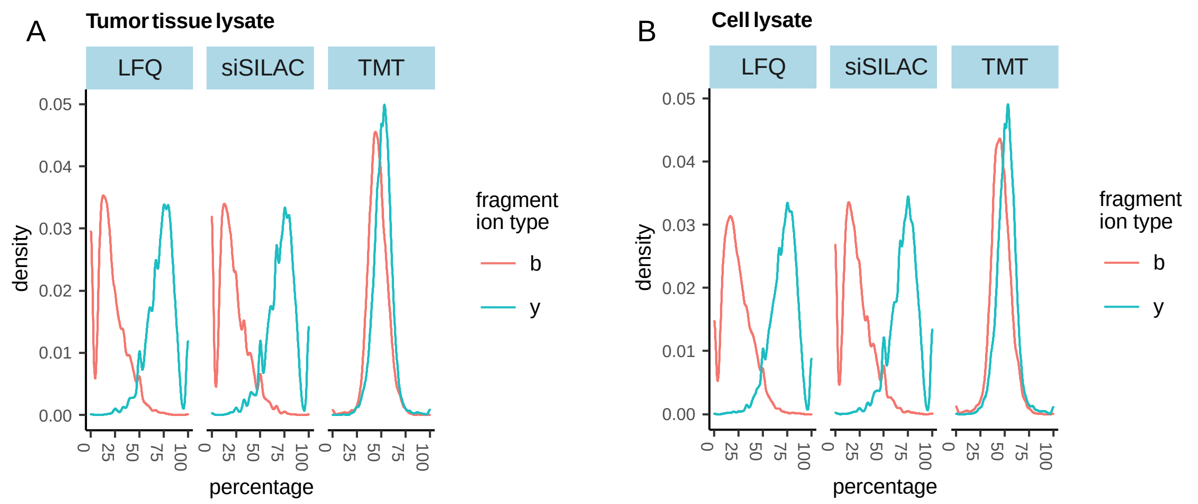

Figure S2: Density plots depicting the distribution of relative b- and y-ion intensity of the identified peptides in tumor tissue lysates (A) and cell lysates (B).

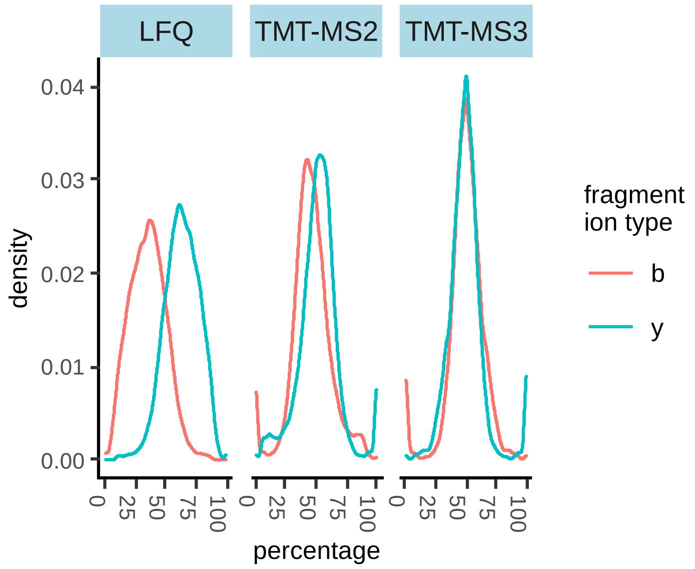

Figure S3: Density plots depicting the distribution of relative b- and y-ion intensity of the identified phosphopeptides in data sets obtained by label-free, TMT-MS2 (HCD fragmentation) and TMT-SPS-MS3 (CID/HCD fragmentation). Re-analysis of HeLa Yeast 1:1 mixture phosphoproteome data from PXD007145 (Hogrebe et al[1]).

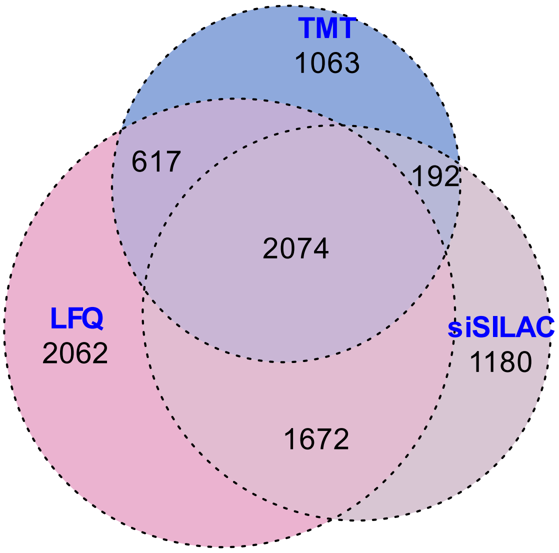

Figure S4: Phosphosite identification with Proteome Discoverer suite using SequestHT and Percolator. Venn diagram showing the number of identified phosphosite by LFQ, spike-in-SILAC (siSILAC) and TMT methods in tumor tissue lysate.

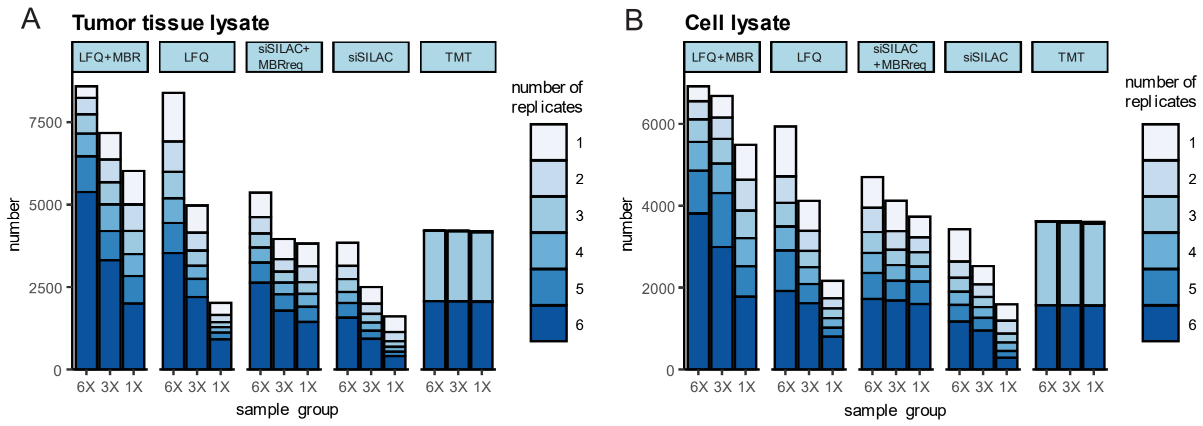

Figure S5: Bar plots showing the number of phosphopeptides identified in each sample group (6X, 3X and 1X) in tumor tissue lysates (A) and cell lysates (B). The color intensity indicates the number of replicates in which the phosphosites were identified.

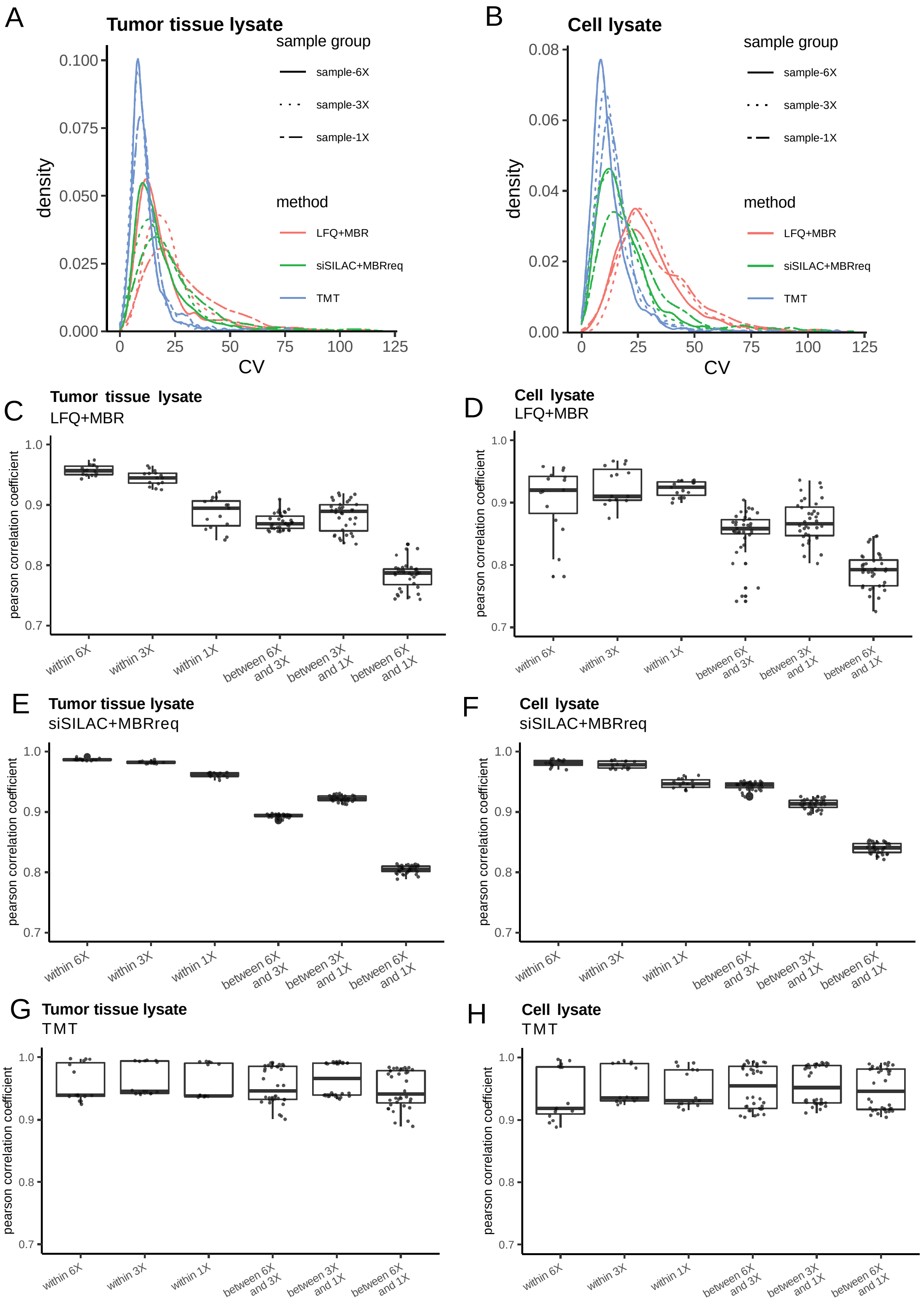

Figure S6: Reproducibility of phosphosite quantification. A, B: Density plots showing the distribution of CV values for phosphosites quantified with LFQ+MBR, spike-in-SILAC+MBRreq and TMT in the tumor tissue lysates (A) and cell lysates (B). Different line types (solid, dotted, dashed) indicate different sample groups (6X, 3X and 1X). C-H: Boxplots visualizing the pairwise correlation coefficients within the same sample group (i.e. 6X vs. 6X, 3X vs. 3X, 1X vs. 1X) and between the different sample groups (i.e., 6X vs. 3X, 3X vs. 1X, 6X vs. 1X) for each method in tumor tissue lysates (C: LFQ+MBR, E: spike-in-SILAC+MBRreq, G: TMT) and cell lysates (D: LFQ+MBR, F: spike-in-SILAC+MBRreq, H: TMT). The boxes show the first, second (median) and third quartile.

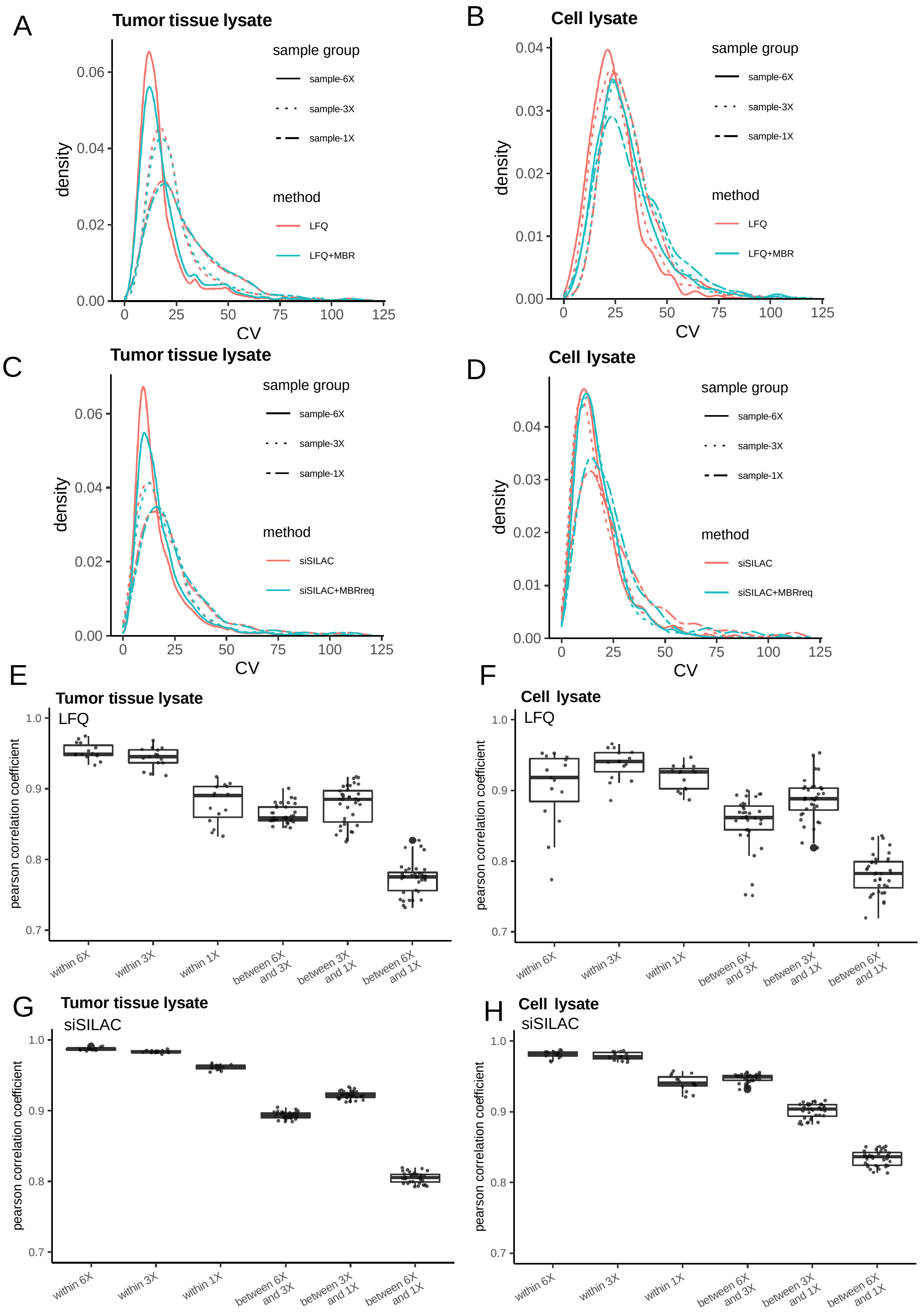

Figure S7: Effect of MBR on the reproducibility of phosphosite quantification. A, B: Density plots showing the distribution of CV values for phosphosites quantified with LFQ+MBR and LFQ in the tumor tissue lysates (A) and cell lysates (B). C, D: Density plots showing the distribution of CV values for phosphosites quantified with spike-in-SILAC + MBRreq and spike-in-SILAC in the tumor tissue lysates (C) and cell lysates (D). Different line types (solid, dotted, dashed) indicate different sample groups (6X, 3X and 1X). E-H: Boxplots visualizing the pairwise correlation coefficients within the same sample group (i.e. 6X vs. 6X, 3X vs. 3X, 1X vs. 1X) and between the different sample groups (i.e., 6X vs. 3X, 3X vs. 1X, 6X vs. 1X) in tumor tissue lysates (E: LFQ, G: spike-in-SILAC) and cell lysates (F: LFQ, H: spike-in-SILAC). The boxes show the first, second (median) and third quartile.

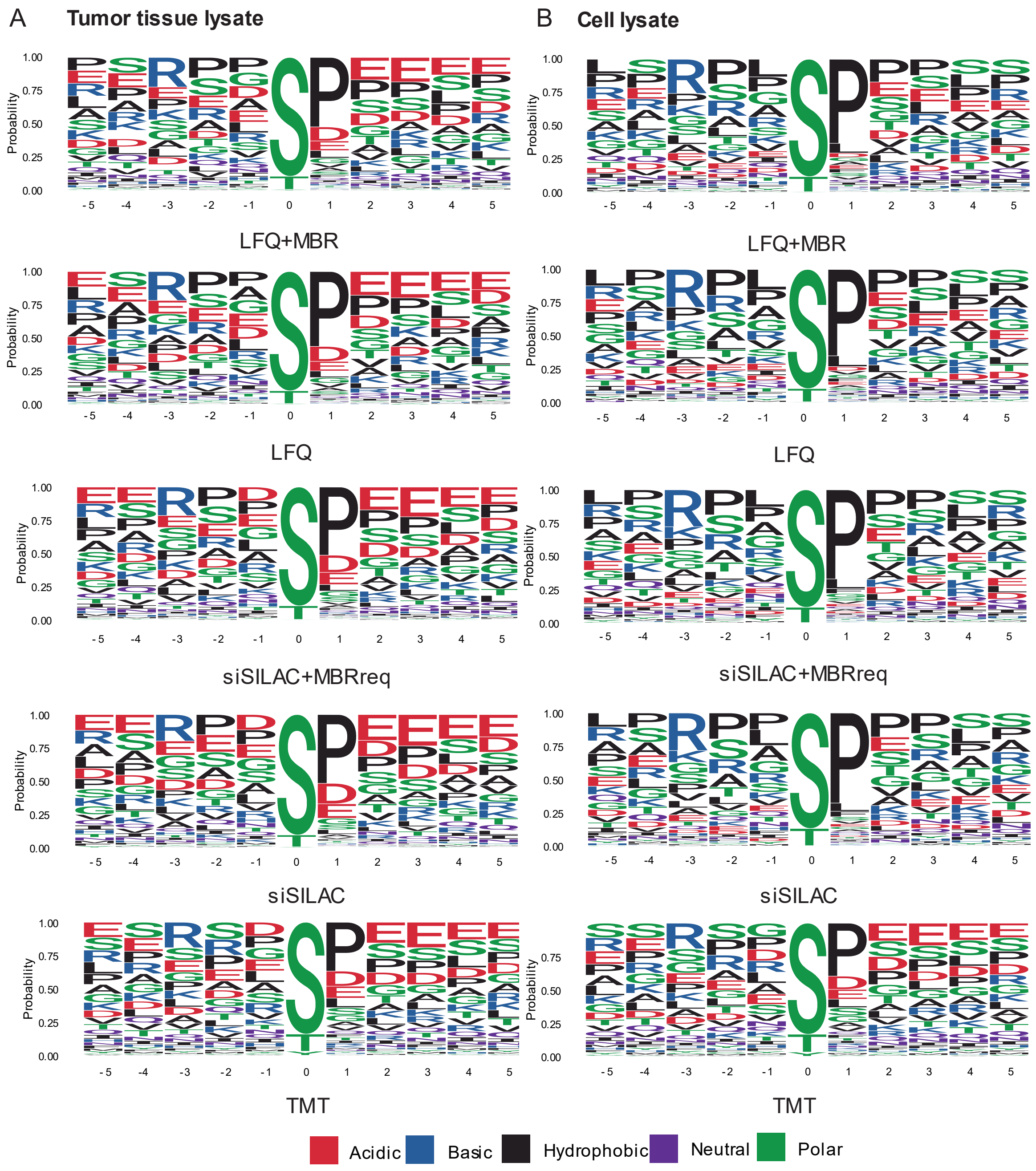

Figure S8: Sequence motif analysis of quantified phosphosites from tumor tissue lysates (A) and cell lysates (B). The phosphorylated residue is located at the central position within a 11-mer phosphorylated peptide sequence. The amino acids are colored according to their chemical properties and the size represents the observed probability.

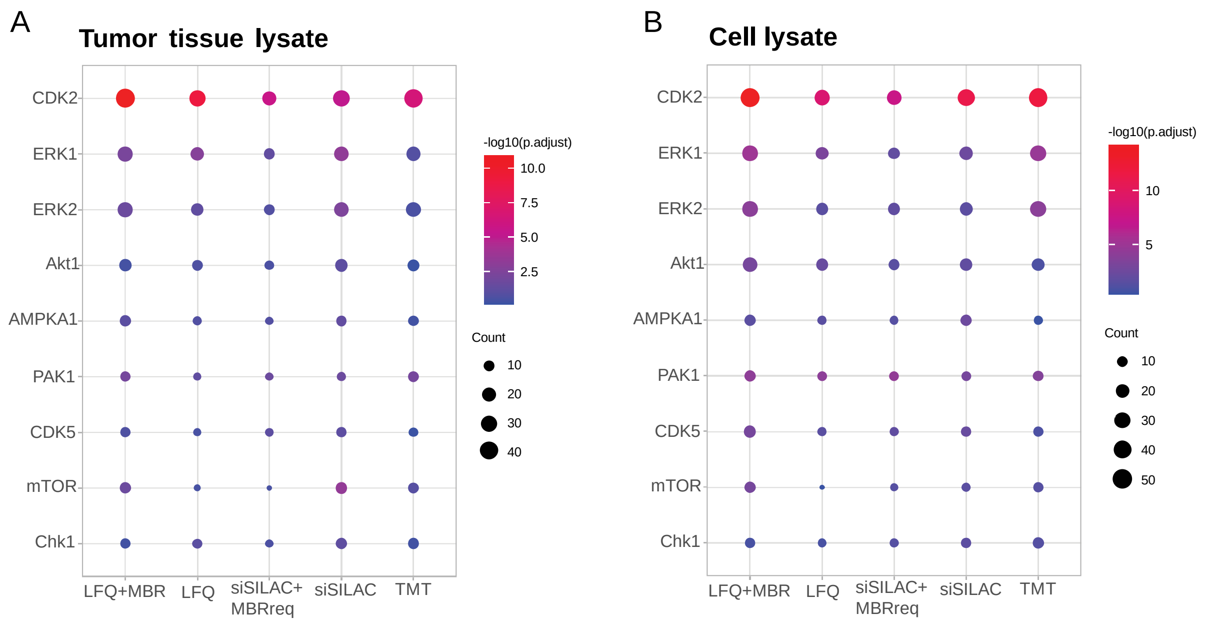

Figure S9: Kinase substrate sites covered by the quantitative phosphoproteomes. Dot-plots visualizing the enrichment analysis of quantified phosphosites from tumor tissue lysates (A) and cell lysates (B). The kinase-substrate database from PhosphositesPlus was used as background dataset. The size of the dots represents the number of phosphosites quantified for the corresponding kinase. The color intensity corresponds to the adjusted p-value using Benjamini-Hochberg approach.

Supplementary tables

	method	measurement time (hour)	number of MS2	number of identified MS2	number of MS3	number of identified phosphopeptides	number of identified unique phosphosites (localization probability > 0.75)	number of identified unique phosphosites per minutes (localization probability > 0.75)
tumor tisse lysate	LFQ	36	471,216	171,753	n.a	8,932	5,812	2.691
	siSILAC	36	613,576	240,688	n.a	6,923	4,282	1.982
	TMT	4	63,421	8,122	63,345	4,282	3,578	29.817
cell lysate	LFQ	36	498,505	206,819	n.a	7,439	4,163	1.927
	siSILAC	36	597,456	236,793	n.a	5,881	3,621	1.676
	TMT	4	56,149	7,059	56,062	3,683	3,023	25.192

Table S3: Key features of tumor tissue and cell phosphoproteome quantified by LFQ, spike-in-SILAC (siSILAC) and TMT with MaxQuant. Measurement time, the number of MS2 spectra, the number of identified MS2 spectra, the number of MS3 spectra, the number of identified phosphopeptides and the number of identified phosphosites with minimum localization probability of 0.75 in the whole dataset or per minute of analysis time.

	method	number of identified unique phosphosites (localization probability > 0.55)	number of identified unique phosphosites (localization probability > 0.75)	number of identified unique phosphosites (localization probability > 0.9)	number of identified unique phosphosites (localization probability > 0.95)
tumor tissue lysate	LFQ	7,726	5,812	4,751	4,299
	siSILAC	5,786	4,282	3,444	3,098
	TMT	4,619	3,578	2,848	2,533
cell lysate	LFQ	5,670	4,163	3,285	2,959
	siSILAC	4,887	3,621	2,873	2,558
	TMT	3,977	3,023	2,345	2,042

Table S4: Number of identified phosphosites with different localization probability thresholds (0.5, 0.75, 0.9 and 0.95).

		phospho-serine		phospho-threonine		phospho-tyrosine	
		number	percentage	number	percentage	number	percentage
tumor tissue lysate	LFQ	5119	88.08%	610	10.50%	83	1.43%
	siSILAC	3813	89.05%	447	10.44%	22	0.51%
	TMT	2961	82.76%	480	13.42%	137	3.83%
cell lysate	LFQ	3655	87.80%	473	11.36%	35	0.84%
	siSILAC	3184	87.93%	409	11.30%	28	0.77%
	TMT	2480	82.04%	428	14.16%	115	3.80%

Table S5: The number and percentage of identified phospho-serine, phospho-threonine and phospho-tyrosine sites with minimum localization probability of 0.75.

	method	measurement time (hour)	number of MS2	number of identified MS2	number of MS3	number of identified phosphopeptides	number of identified unique phosphosites (localization probability > 0.75)	number of identified unique phosphosites per minutes (localization probability > 0.75)
tumor tisse lysate	LFQ	36	426,862	177,548	n.a	8,556	6,425	2.975
	siSILAC	36	579,022	234,623	n.a	6,072	5,118	2.369
	TMT	4	57,537	9,846	63,345	4,964	3,946	32.883

Table S6: Key features of tumor tissue phosphoproteome quantified by LFQ, spike-in-SILAC (siSILAC) and TMT with Proteome Discoverer suite using SequestHT and Percolator. Measurement time, the number of MS2 spectra, the number of identified MS2 spectra, the number of MS3 spectra, the number of identified phosphopeptides and the number of identified phosphosites with minimum localization probability of 0.75 in the whole dataset or per minute of analysis time.

	tumor tissue lysate					
	true positive rate			AUROC		
fold change	FC2	FC3	FC6	FC2	FC3	FC6
LFQ+MBR	0.7500	0.9700	0.9900	0.9394	0.9852	0.9938
LFQ	0.9900	0.9940	0.9990	0.9953	0.9971	0.9990
siSILAC+MBRreq	0.7700	0.8800	0.9800	0.9442	0.9633	0.9899
siSILAC	0.9200	0.9700	0.9900	0.9702	0.9881	0.9960
TMT	0.9900	0.9970	0.9980	0.9942	0.9988	0.9990
	**cell lysate**					
	true positive rate			AUROC		
fold change	FC2	FC3	FC6	FC2	FC3	FC6
LFQ+MBR	0.5990	0.9400	0.9690	0.8923	0.9749	0.9916
LFQ	0.5910	0.9200	0.9700	0.8992	0.9813	0.9900
siSILAC+MBRreq	0.9300	0.9000	0.9950	0.9817	0.9673	0.9931
siSILAC	0.9500	0.9800	0.9970	0.9815	0.9925	0.9988
TMT	0.9680	0.9930	0.9990	0.9879	0.9933	0.9985

Table S7: True-positive-rates (TPRs) for the different quantification methods at a false-positive-rate (FPR) threshold of 0.05, and the area under the receiver operating characteristic (AUROC).

Supplemental experimental section

SKOV3 cell culture and SILAC labeling. The SKOV3 cells were purchased from ATCC (HTB-77, Homo sapiens ovary). Cells were cultivated in lysine- and arginine-free Dulbecco's modified Eagle's medium (1111DMEM) with 1 g/L glucose, (PAN Biotech, cat. no. P04-02506), supplemented with 10% (v/v) dialyzed fetal bovine serum (FBS) (Gibco, cat. no. 10270-106), D-(+)-Glucose solution (Sigma-Aldrich, cat. no. G8644-100 ML) to a final concentration of 4.5 g/L, 3 mM L-glutamine (Gibco, cat. no. 25030-024), 84 mg/L lysine and 146 mg/L arginine. For SILAC labeling "heavy" lysine ($^{13}C6$, $^{15}N2$, Cambridge Isotope Laboratories Inc., CNLM-291-H-PK) and arginine ($^{13}C6$, $^{15}N4$, Cambridge Isotope Laboratories Inc., CNLM-539-H-PK) (heavy labeled medium) or "light" lysine (Cambridge Isotope Laboratories Inc., ULM-8766-PK) and arginine (Cambridge Isotope Laboratories Inc., ULM-8347-PK)[2] (light labeled medium) was used. For TMT and LFQ analysis cells cultivated in light labeled medium were used. All cells were maintained at 37ºC and 7.5% CO_2 for at least five passages and regularly tested for mycoplasm. Arginine-to-proline conversion and SILAC labeling efficiency was tested by LC-MS/MS. The arginine-to-proline conversion was at or below 1% and the SILAC labeling efficiency was higher than 98%.

SKOV3 cell line and ovarian cancer tissue lysis. For serum and amino acid starvation, SKOV3 cells were washed with phosphate-buffered saline (PBS, PAN Biotech, cat. no. P0436500) and cultured for 16 h in amino acids free DMEM medium (PAN Biotech, cat. no. P0401507). For stimulation, the medium was exchanged to light labeled medium for 90 min. Cells were washed three times with ice-cold PBS and lysed in protein lysis buffer [8 M urea, 75 mM NaCl, 50 mM Tris, pH 8.2] supplemented with Complete Mini (Roche, cat. no. 04 693 124 001) and PhosSTOP (Roche, cat. no. 04 906 845 001).

The ovarian cancer tissue was provided by PROMETOV and analyzed under the ethics board approval S496/2014. For tissue lysis, frozen tumor samples were crushed on dry ice and subsequently pulverized at 35 Hz for 2 min in liquid nitrogen precooled teflon milling cups using a ball mill (Retsch Mixer Mill MM 400) and a metal ball. The well-mixed tissue powder was aliquoted on dry ice before taking up in protein lysis buffer.

Cell lysates and tissue lysates were sonicated at 20% amplitude with a 20 x 1 sec pulse on ice (1 sec sonication and 1 sec cooling down), before centrifugation at 12,500 × g, 4°C for 10 min. The supernatant was transferred to new tubes. The protein concentrations were measured using the BCA Protein Assay Kit (Thermo-Fisher Scientific, cat.no. 23235) according to the manufacturer's protocol and adjusted to 1 mg/mL.

Protein reduction, alkylation and digestion. To reduce disulfide bonds, lysates were incubated with 5 mM DTT at 37°C under gentle shaking for 1 h. To alkylate cysteine residues, iodoacetamide was added to a final concentration of 14 mM and incubated for 30 min at RT in the dark. Subsequently, DTT was added to a final concentration of 5 mM and the samples were incubated for

an additional 15 min at RT in the dark. Next, the samples were diluted with a ratio of 1:5 with 25 mM Tris-HCl, pH 8.2 to reduce the urea concentration to 1.6 M. For tryptic digestion, the samples were incubated with trypsin (trypsin to substrate ratio of 1:50, Promega, cat. no. V5111) and 1 mM $CaCl_2$ for 16 h at 37°C. The tryptic digestion was stopped by adding TFA to a final concentration of 0.4% (v/v), respectively. The samples were desalted using SepPak tC18 cartridges (Sep-Pak tC18 1 cc Vac Cartridge, 100 mg Sorbent per Cartridge, 37 - 55 µm, cat. no. WAT036820). The cartridges were conditioned using two times 1 mL condition buffer (95% ACN, 5% H_2O, 0.1% TFA), before washing two times with 1 ml of wash buffer (5% ACN,95% H_2O, 0.1% TFA). Samples were loaded to the cartridges followed by washing three times with 1 mL wash buffer. The peptides were eluted using two times 1 mL elution buffer (50% ACN, 50% H_2O). For the LFQ and spike-in-SILAC measurements, 50 µL of the eluent was used for the LC-MS/MS analysis and 950 µL for the subsequent IMAC enrichment. All the samples were dried using a SpeedVac.

LC-MS/MS measurement. For LC-MS/MS analysis, samples were injected on an ultrahigh performance nano liquid chromatography system (Dionex UltiMate 3000 RSLCnano, Thermo Scientific, Bremen, Germany) coupled to an Orbitrap mass spectrometer (Fusion™, Thermo Fisher Scientific) with a nano electrospray source.

The samples were loaded (3 µL/min) with the buffer A (0.1% formic acid (FA) in HPLC grade H_2O) on a trapping column (Acclaim PepMap µ-precolumn, C18, 300 µm × 5 mm, 5 µm, 100 Å, Thermo Scientific, Bremen, Germany). After sample loading, the trapping column was washed with 30 µL buffer A (3 µL/min) and the peptides were eluted (300 µL/min) onto separation column (Acclaim PepMap 100, C18, 75 µm × 500 mm, 2 µm, 100 Å, Thermo Scientific, Bremen, Germany). The column temperature was kept constant at 45°C and the peptides were separated with a gradient from 5−25% buffer B in 90 min. The spray was generated from a silica emitter with conductive coating (O.D. 360 µm, I.D. 20 µm, Tip I.D. 10 µm New Objective, Littleton, USA) at a capillary voltage of 1800 V. MS analyses were performed in positive ion mode and data-dependent acquisition mode (DDA). LC-MS/MS analysis was carried out in a cycle time of 3 s and the dynamic exclusion duration was set to 30 s. For the spike-in-SILAC and LFQ samples, an intensity threshold of $2{\cdot}10^4$, an isolation width of 0.7 m/z and an HCD collision energy of 30 NCE was used for MS/MS experiments. Precursor MS scans were performed over a m/z range from 380-1500, with a resolution of 120,000 FWHM at m/z 200 (RF Lens = 60%, maximum injection time= 50 ms, AGC target= $2{\cdot}10^5$) and MS/MS spectra were recorded with a resolution of 7,500 FWHM at m/z 200 (maximum injection time= 22 ms, AGC target= $2{\cdot}10^5$).

For TMT, we used multi-notch synchronous precursor selection (SPS)-MS3 technology to co-isolate and co-fragment multiple MS2 fragment ions ion the linear ion trap, followed by reporter ions detection in the orbitrap (MS3). The SPS-MS3 approach reduces reporter ion variance and enhance quantification accuracy[3,4]. An intensity threshold of $5{\cdot}10^3$ and an isolation width of 0.7 m/z was used for MS/MS experiments. Precursor MS scans were performed over a m/z range from 380-1500, with

a resolution of 120,000 FWHM at m/z 200 (RF Lens = 60%, maximum injection time= 50 ms, AGC target = $2 \cdot 10^5$) and the precursors were fragmented by collision-induced dissociation (CID) (AGC $1 \cdot 10^5$, normalized collision energy (NCE) = 35, q-value = 0.25, maximum injection time = 50 ms, isolation window = 0.7) and measured in the ion trap (ion trap scan rate = turbo). For each MS2 spectrum, we collected an MS3 spectrum in which multiple MS2 fragment ions are captured using isolation waveforms with multiple frequency notches. MS3 precursors were fragmented by HCD and analyzed using the Orbitrap (NCE = 65, AGC = $1 \cdot 10^5$, maximum injection time = 120 ms, resolution = 60,000, m/z range = 120-500).

Raw data processing. All LC-MS/MS data were processed with MaxQuant[5] version 1.6.5 or Proteome Discoverer suits. Peptides and proteins identification was performed using the Andromeda search engine[6] (MaxQuant) or SequestHT with Percolator (Proteome Discoverer) and searched against the Swissprot database (Uniprot, downloaded 2019-09-20, 20430 entries) and a contamination database (cRAP-database, http://www.thegpm.org/crap, 298 entries)[7]. For both analyses with Proteome Discoverer and MaxQuant, FDRs were calculated using a decoy database. In both approaches, the decoy databases were generated by inverting all protein sequences. Carbamidomethylation of cysteines was specified as fixed modification and oxidation of methionine, and N-terminal protein acetylation and phosphorylation of serine, threonine and tyrosine residues were defined as variable modifications. TMT correction factors were added in MaxQuant or Proteome Discoverer using the values provided by the reagent manufacturer. Only phosphopeptides/phosphosites with a localization probability larger than 0.75 in MaxQuant or ptmRS site probabilities above 75% in Proteome Discoverer were considered for further analysis. In Proteome Discoverer, only those peaks with a S/N threshold higher than 1.5 were used to search against to the database. In Maxquant, for analysis of LFQ measurements, data were searched with and without "match between runs" (MBR). For analysis of the spike-in-SILAC measurements, the data were searched with and without MBR and re-quantify (req) option.

Data reanalysis from Hogrebe et al.[1] was performed by downloading HeLa and Yeast phosphopeptide dataset from ProteomeXchange with identifier of PXD007145 using the following files: LFQ (20171106_LUMOS1_nLC13_AH_TechBench2_LFQ_1_1, 20171106_LUMOS1_nLC13_AH_TechBench2_LFQ_1_3, 20171106_LUMOS1_nLC13_AH_TechBench2_LFQ_1_2), TMT-MS2 (20170328_LUMOS1_nLC13_AH_TMTYoccHback_MS2_1, 20170328_LUMOS1_nLC13_AH_TMTYoccHback_MS2_2, 20170328_LUMOS1_nLC13_AH_TMTYoccHback_MS2_3), TMT-SPSMS3 (20170328_LUMOS1_nLC13_AH_TMTYoccHback_MS3_1, 20170328_LUMOS1_nLC13_AH_TMTYoccHback_MS3_2, 20170328_LUMOS1_nLC13_AH_TMTYoccHback_MS3_3).

Reference

(1) Hogrebe, A.; Von Stechow, L.; Bekker-Jensen, D. B.; Weinert, B. T.; Kelstrup, C. D.; Olsen, J. V. Benchmarking Common Quantification Strategies for Large-Scale Phosphoproteomics. *Nat. Commun.* **2018**, *9* (1). https://doi.org/10.1038/s41467-018-03309-6.

(2) Ong, S. E.; Blagoev, B.; Kratchmarova, I.; Kristensen, D. B.; Steen, H.; Pandey, A.; Mann, M. Stable Isotope Labeling by Amino Acids in Cell Culture, SILAC, as a Simple and Accurate Approach to Expression Proteomics. *Mol. Cell. Proteomics* **2002**, *1* (5), 376–386. https://doi.org/10.1074/mcp.M200025-MCP200.

(3) McAlister, G. C.; Nusinow, D. P.; Jedrychowski, M. P.; Wühr, M.; Huttlin, E. L.; Erickson, B. K.; Rad, R.; Haas, W.; Gygi, S. P. MultiNotch MS3 Enables Accurate, Sensitive, and Multiplexed Detection of Differential Expression across Cancer Cell Line Proteomes. *Anal. Chem.* **2014**, *86* (14), 7150–7158. https://doi.org/10.1021/ac502040v.

(4) Ting, L.; Rad, R.; Gygi, S. P.; Haas, W. MS3 Eliminates Ratio Distortion in Isobaric Multiplexed Quantitative Proteomics. *Nat. Methods* **2011**, *8* (11), 937–940. https://doi.org/10.1038/nmeth.1714.

(5) Cox, J.; Mann, M. MaxQuant Enables High Peptide Identification Rates, Individualized p.p.b.-Range Mass Accuracies and Proteome-Wide Protein Quantification. *Nat. Biotechnol.* **2008**, *26* (12), 1367–1372. https://doi.org/10.1038/nbt.1511.

(6) Cox, J.; Neuhauser, N.; Michalski, A.; Scheltema, R. A.; Olsen, J. V.; Mann, M. Andromeda: A Peptide Search Engine Integrated into the MaxQuant Environment. *J. Proteome Res.* **2011**, *10* (4), 1794–1805. https://doi.org/10.1021/pr101065j.

(7) Mellacheruvu, D.; Wright, Z.; Couzens, A. L.; Lambert, J.; St-denis, N.; Li, T.; Miteva, Y. V; Hauri, S.; Sardiu, M. E.; Yew, T.; Halim, V. A.; Bagshaw, R. D.; Hubner, N. C.; Bouchard, A.; Faubert, D.; Fermin, D.; Dunham, W. H. HHS Public Access. *Nat Methods.* **2014**, *10* (8), 730–736. https://doi.org/10.1038/nmeth.2557.The.
